# Switching from Infliximab to Biosimilar in Inflammatory Bowel Disease: A Review of Existing Literature and Best Practices

**DOI:** 10.1093/crocol/otaa093

**Published:** 2021-02-15

**Authors:** Shubha Bhat, Taha Qazi

**Affiliations:** 1 Department of Pharmacy, Crohn’s and Colitis Program, Boston Medical Center, Boston, Massachusetts, USA; 2 Department of Gastroenterology, Crohn’s and Colitis Program, Boston Medical Center, Boston, Massachusetts, USA; 3 Department of Gastroenterology, Hepatology & Nutrition, Digestive Diseases Institutes, Cleveland Clinic, Cleveland, Ohio, USA

**Keywords:** biosimilars, switch, infliximab, inflammatory bowel disease

## Abstract

Biosimilars are highly similar but nonidentical biologic agents with no differences in clinical efficacy and safety when compared to bio-originator products. Considering the long-term costs of managing inflammatory bowel disease (IBD), biosimilars, through economic competition, provide an opportunity for cost savings to payors, may increase access to IBD medications, and could decrease health care spending in the long run. Studies investigating the use of biosimilars in IBD have shown a comparable clinical efficacy and safety profile compared to originator products. Moreover, studies have also suggested that solitary switches between bio-originators and biosimilars are acceptable and do not lead to worsening disease burden or increased immunogenicity or safety concerns. Despite available data and proposed benefits of biosimilars, skepticism about the widespread adoption of biosimilars throughout the United States continues to be present and creates many barriers. Herein, we detail the real-world, nonmedical switching experiences of 2 IBD centers in the United States and review best practices, which can be used as a potential roadmap for successful biosimilar adoption in other institutions.

## INTRODUCTION

Chronic inflammatory bowel diseases (IBDs), including Crohn’s disease and ulcerative colitis, are relapsing, remitting conditions that affect the gastrointestinal tract. One approach to managing these conditions is the use of biologic therapies, specifically monoclonal antibodies, to modulate the immune system and control inflammation. Infliximab, a monoclonal antibody directed against tumor necrosis factor α (TNF-α), is an efficacious treatment for both moderate to severe Crohn’s disease and ulcerative colitis.^1,2^ It is also the first Food and Drug Administration (FDA)-approved biologic for the management of IBD and remains a first-line treatment option.^3,4^

Biologics such as infliximab represent only 2% of drug prescriptions, but in 2017, accounted for 38% of drug spending.^5^ Moreover, spending on biologic therapies has grown 10.7% annually from 2014 to 2018 (IQVIA). In the biologic era, the burden of costs that stems from IBD management has shifted from inpatient and operative management to pharmaceutical-based costs of care.^6,7^ A means to manage this rising cost, such as the use of biosimilars, is a pertinent area of focus in controlling health care spending.

Biosimilars are biological products that are highly similar to the bio-originator product, but not considered identical due to molecular complexity. As such, biosimilars cannot be classified as “generics,” which are clinically synthesized, small molecule compounds that are identical to the reference product. Biosimilars also often have minor differences in components that are clinically inactive compared to the reference product, but, overall, are not different in regards to pharmaceutical function or safety. For FDA approval, biosimilars must undergo an abbreviated drug application pathway known as the 351(k), which was created by the Biologics Price Competition and Innovation Act. In this pathway for biosimilar approval, manufacturers are required to demonstrate similarity with the bio-originator in terms of structure, function, toxicity, pharmacokinetics, and pharmacodynamics. With this criteria, an expedited process of approval occurs using the totality of evidence. Additionally, based on the data provided to the FDA, the agency can deem extrapolation of indication as appropriate; as a result, biosimilars are approved for all indications that the bio-originator is approved for, including IBD.

Several biosimilars are currently FDA-approved for the management of IBD. These include infliximab biosimilars: infliximab-dyyb (Celltrion), infliximab-abda (Samsung Bioepsis/Merck), and infliximab-axxq (Amgen). Adalimumab, another monoclonal antibody directed against TNF-α, is also produced as a biosimilar by many companies, including adalimumab-atta (Amgen), adalimumab-adbm (Boehringer Ingelheim), adalimumab-adaz (Sandoz), and adalimumab-afzb (Pfizer). Compared to the bio-originator product, biosimilars are priced lower.

As thus, the rationale for switching from a bio-originator product to a biosimilar is largely based on cost benefits. Increasing competition between biosimilars and bio-originator products results in decreasing costs for payors and with long-term use, can lead to decreases in overall health care spending. Moreover, the introduction of several biosimilar products into the market can help increase patients’ access to medications. Switching between bio-originator and biosimilar products is however not a simple process and involves detailed coordination and acceptance from several stakeholders. With proper strategies, biosimilar adoption can be a successful endeavor.

Herein we describe the stepwise approaches and successful experiences of switching from bio-originator to biosimilar products at both the Boston Medical Center (BMC) and Cleveland Clinic Foundation (CCF). We also review the opportunities, threats, and potential controversies surrounding biosimilar adoption and current best practices for biosimilar switching.

## BIOSIMILAR ADOPTION PROCESS AND EXPERIENCES

### BMC Model

BMC is the largest safety-net academic institution in the New England area, with 841,000 outpatient visits occurring across primary and specialty clinics annually. In fall 2017, the pharmacy department, driven by cost-saving initiatives, explored the idea of adopting infliximab-dyyb onto formulary with affected stakeholders from dermatology, gastroenterology, and rheumatology settings. Various providers from these settings initially expressed reservation, primarily stemming from concerns of indication extrapolation, appropriate timeframe for biosimilar switch, unclear benefit to patients from a financial perspective, and interruption in patients’ infusion experiences. A pharmacy team, consisting of a clinical pharmacist and certified pharmacy technician, embedded in the gastroenterology practice took the lead in addressing providers’ concerns by gathering clinical evidence, designing educational teaching points about benefits of biosimilar switch including the potential for lower premiums, and creating a workflow to ensure a seamless switch process. Additionally, criteria for infliximab-dyyb transition were created and only patients who were on infliximab at least 6 months, approved by their provider to transition, provided consent, covered by their payor to receive infliximab-dyyb, and received infliximab infusion at BMC were eligible to switch to infliximab-dyyb. With these interventions, provider buy-in was obtained.

Infliximab-dyyb was approved by the Pharmacy and Therapeutics committee and added to the formulary in December 2017. To ensure adequate time for the pharmacy team to gather a list of eligible patients and conduct outreach, the biosimilar nonmedical switch process began in March 2018. The operational details and outcomes of the biosimilar switch process have been previously described.^8^ In summary, the pharmacy team drove the biosimilar switch process, from conferring with providers which patients to switch, obtaining insurance approval via prior authorizations, contacting the patient to provide education on infliximab-dyyb and need for consent, and if consent was obtained, ensuring that the patient received infliximab-dyyb at the next infusion appointment. The last step required extensive coordination between the gastroenterology pharmacist, patient’s provider, and infusion center staff, including pharmacists and nurses. With this process, 97% of patients were successfully switched to infliximab-dyyb. Within the cohort of patients with IBD who switched, no clinical differences were observed.

### Cleveland Clinic Model

The Cleveland Clinic Health System is an academic medical foundation, incorporating a large, medical center in Cleveland, as well as 11 regional hospitals in northeast Ohio. Nationally, the CCF also operates 5 hospitals in southeast Florida and a medical center for brain health in Las Vegas. Internationally, the Cleveland Clinic Health System also includes a hospital in Abu Dhabi, a sports and executive health center in Toronto, Ontario, as well as a planned opening of a hospital in London.

As an opportunity to improve specialty drug costs, the supply chain team at the Cleveland Clinic placed a Request for Proposal (RFP) in March 2018 to all manufacturers of infliximab biosimilars as well as the reference product. Through the RFP, the Cleveland Clinic pharmacy administrative practice investigated and was able to directly negotiate with the leading pharmaceutical companies regarding the exclusive provision of biologic therapies across the institution. In conjunction, this RFP was combined by a payor market review to ensure that the insurers of patients managed with the potential selected biosimilars were covered following the transition. Following negotiations, Merck pharmaceuticals was selected as the potential purveyor of adult biologic therapies. Specifically, the biosimilar, infliximab-abda, became the preferred anti-TNF therapy for the treatment of immune-mediated inflammatory disorders, including ulcerative colitis and Crohn’s disease at the Cleveland Clinic and affiliated hospitals and health centers. A notable exclusion was the Cleveland Clinic Children’s Institute, which was not included in the transition process. The conversion process was reviewed and approved by the Cleveland Clinic Health Systems Pharmacy and Therapeutics Committee, the governing body for the formulary at Cleveland Clinic.

Following the selection of infliximab-abda, the pharmacy administration met with the chairs from the primary departments providing anti-TNF therapy, including the Digestive Disease Institute, Orthopedic and Rheumatologic Institute, Respiratory Institute, and the Dermatology and Plastic Surgery Institute. The purpose of the discussion was not only to provide the current knowledge on anti-TNF biosimilar therapy but also to allay concerns regarding the possible switch. Following initial discussions, institute leaders were able to communicate the plan for potential transition and switching to individual providers within institutes. As a part of these conversations, opportunities, barriers, and threats of biosimilar adoption were identified. Leaders in the pharmacy administrative practice led discussions with institute leaders to assuage apprehensions during these conversations.

After these discussions, January 2, 2019 was identified as the start date for the institute-wide, nonmedical switch in therapy. The period between initial discussions and switch provided adequate time for providers to introduce and approach patients on medical therapy regarding switching. Additionally, educational materials and information regarding biosimilars were made available to patients, who had time to discuss the potential switch with providers. With patient and provider synergy, a successful switch of patients was implemented across the CCF. Like BMC, certain patients with insurance payors who were unable to cover infliximab-abda were maintained on the bio-originator therapy.

Although data from the switch are pending, in the first 9 months, CCF was able to successfully convert 2711 infusions out of 2936 to the biosimilar, infliximab-abda, across several indications for immune-mediated inflammatory disorders. Of the patients unable to receive infliximab-abda, 71% were due to payor restriction in covering the biosimilar. The remainder were adult patients treated by pediatric providers and were continued on the bio-originator. Data from the adverse event reporting across 6 months following conversion demonstrated only 22 adverse events, of which none were documented in patients with IBD.

## LESSONS LEARNED FROM TRANSITIONS

Although BMC and CCF are 2 separate entities and independently designed and led the biosimilar transition process, common barriers and approaches to overcome these barriers were present in both experiences. First, provider buy-in was critical to formulary adoption and reassuring patients about the transition. Some providers, particularly gastroenterologists, were concerned about indication extrapolation, efficacy and safety data available in IBD, and immunogenicity following the switch. Several studies, including the NOR-SWITCH trial, are summarized in Table 1 and were reviewed with gastroenterologists to demonstrate that the infliximab-dyyb and infliximab-abda switch is safe and effective for patients. Additionally, data from unpublished and presented abstracts were also reviewed. The data on these switches are summarized in Table 2. By involving stakeholders early in the process and providing them with a platform to express any concerns, we were able to obtain full buy-in and implement additional processes in place to monitor the efficacy and safety of biosimilar switching in real-world settings.

**TABLE 1.** Published Switching Studies Performed in Patients With Inflammatory Bowel Disease

Type of Switch	Definition	Results
Bio-originator to biosimilar switch	Single switch from bio-originator to biosimilar	Several studies demonstrating noninferiority of switching compared to staying on bio-originator in terms of clinical disease scores, CRP, disease activity, or long-term clinical remission^9–11^ Large-scale database study also suggestive of similar efficacy and safety profile of biosimilar compared to bio-originator^12^ No significant difference in drug levels at baseline compared to post-switch^13^ No significant difference in the development of anti-drug antibodies in patients switched compared to those not switched. Antidrug antibodies in biosimilar and bio-originator products demonstrated full cross-reactivity^14^
Reverse switching	Single switch from a biosimilar product to a bio-originator product	174 patients with IBD (136 with CD and 38 with UC)^15^ • Switched from biosimilar to bio-originator • No significant difference in clinical remission following switch at weeks 16, 24 • Serum drug levels similar before and after switch with no significant difference in anti-drug antibodies

CD, Crohn’s disease; CRP, C-reactive protein; IBD, inflammatory bowel disease; UC, ulcerative colitis.

**TABLE 2.** Unpublished Switching Studies Performed in Patients With Inflammatory Bowel Disease (Abstract Presentation)

Type of Switch	Definition	Results
Cross switch	Single switch between biosimilar products	133 patients with IBD (105 with CD and 28 UC) on maintenance biosimilar switched to different biosimilar^16^ • No significant difference between clinical disease scores in switched patients from baseline compared to weeks 16/18 after switch • No significant difference in drug persistence compared to the historical cohort. 221 patients (179 CD and 42 UC) switched between biosimilars^17^ • Increase in drug trough level following switch • No difference in CRP and no new antidrug antibodies detected
Double switch/second switching	Switching twice between biosimilar and/or bio-originator products	52 patients with IBD double switched (39 CD and 13 UC)^18^ • Majority of patients in clinical remission (94%) at week 24 following the second switch • 86% remained on infliximab therapy following double switch • No difference in clinical remission, infliximab discontinuation, and adverse events in single vs double switched patients. Single switch (57 CD and 30 UC) compared to double switch (95 CD and 4 UC)^17^ • No significant difference in clinical disease scores or CRP in patients singly switched vs double switched

CD, Crohn’s disease; CRP, C-reactive protein; IBD, inflammatory bowel disease; UC, ulcerative colitis.

From a patient perspective, at BMC, most patients were reachable via telephone and amenable to the switch; alternatively, unreachable patients could be captured at the infusion center. Given patients’ lack of knowledge regarding biosimilars,^12^ education was provided by the pharmacist. Before providing consent, a few patients wished to speak with their providers, which required additional time from the providers. In an effort to protect provider time in the process of biosimilar conversion several proactive steps can be undertaken. These include: 1) Having a pharmacy team spearhead the conversion 2) Disseminating educational materials to patients when possible 3) Providing one month for providers to proactively mention the biosimilar switch during their patient encounters, and 4) Involving infusion unit nurses to inform patients about the transition.

Lastly, some payors posed as a barrier. Rebate agreements between pharmaceutical companies, benefit managers, and payors may create incentives for bio-originator products over biosimilars. Rebates can significantly decrease payor-related costs, but may be revoked if payors adopt biosimilars, thereby resulting in higher net costs to payors, known as the “rebate trap.” Certain payors refused to cover infliximab-dyyb or infliximab-abda, stating that patients must fail the reference product before switch, which is not a clinically appropriate action. As thus, patients covered by these payors remained on reference infliximab.

### Pathway to Transition

**FIGURE 1.**
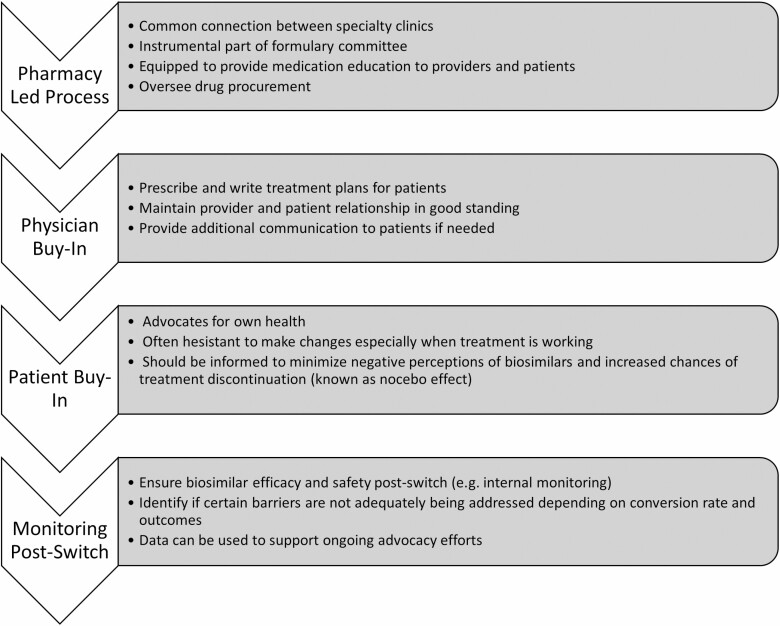
Key steps to incorporate in biosimilar switching and importance.

Key steps utilized in the biosimilar switching process by both BMC and CCF are summarized in Figure 1. Best practices derived from these experiences are detailed in Table 3. To successfully overcome barriers to biosimilar switching, these steps and best practices should be integrated into the process utilized by institutions looking to promote biosimilar adoption and use.

**TABLE 3.** Best Practices for Nonmedical Biosimilar Switching

• All involved stakeholders (providers, nurses, pharmacists, administration) should convene to discuss concerns, review existing data, and come to a consensus about biosimilar switching. • The pharmacy team, if available, is best suited to drive the biosimilar adoption and transition process. The team can assist with the presentation to the Pharmacy and Therapeutics (P&T) Committee for approval to add biosimilar onto formulary, provide biosimilar education to the staff and patients, oversee the transition process, and monitor real-world outcomes post-transition. If a pharmacy team is not available, one team should be designated to handle these tasks. • Education materials should be created and disseminated to patients. Patients should have conversations with the staff to address biosimilar questions or concerns. With a shared decision-making process, patients’ consent to switch should be obtained. • The process of biosimilar adoption and transition should be operationalized and consistent across the institution. • Follow-up to address patients’ concerns and monitor real-world outcomes should be provided. • Advocacy efforts to overcome additional barriers to biosimilar adoption and transition should be undertaken.

## ONGOING BARRIERS LIMITING USE OF BIOSIMILARS

Although BMC and CCF were able to convert most patients, a biosimilar switching rate of 100% was not feasible due to ongoing barriers, which are currently being addressed by additional approaches or advocacy efforts. The guidance from all the major gastroenterology societies reports that the use of a biosimilar in biologic-naïve patients is supported by data and is situationally suitable for novel starts of therapy. This is the guidance from both position statements by both European societies, including European Crohn’s and Colitis Organization, the British Society of Gastroenterology, the European Society of Pediatric Gastroenterology Hepatology and Nutrition and American Societies, including the Crohn’s and Colitis Foundation, Crohn’s Colitis Canada, and American Gastroenterological Association.^19–21^ Society position statements also recommend that medical switching to biosimilars should be individualized and patients should be informed on the decision-making process.

Interchangeability is an FDA designation that allows the substitution of a biosimilar without the intervention of the prescribing health care provider. The FDA has not defined the evidence needed for interchangeability, but additional data regarding multiple switches with a demonstration of no safety or efficacy differences between bio-originator and biologic products are likely required. Due to the lack of this designation, automatic switching is not recommended by the major gastroenterology societies. As additional studies emerge involving switches, the designation may be granted. However, this issue is legally controversial and is being debated at state levels in the United States.

Nonmedical switching, defined as switching to biosimilars guided by nonmedical reasons, is generally condoned by major societies. This is primarily based on the aforementioned studies where mandated by cost pressures requiring switching or reverse switching. Nevertheless, Crohn’s Colitis Canada has recommended against nonmedical switching.^20^ These differences in society recommendations result in variation in practice regarding switches and add to the uncertainty in the use of biosimilar agents. Nevertheless, as providers become more familiar with biosimilars and patient education around biosimilar agents matures, a unified statement from major societies may remedy these variations in practice.

From a payor perspective, the major barriers include rebate mechanisms provided by pharmaceutical companies, which in turn allows the payors to benefit from cost savings by covering only the reference product, rather than the biosimilar.^22^ As a result, patients are maintained on the reference product. Formulary exclusivity refers to an anachronistic concept where patients are required to fail a bio-originator product before being approved for a biosimilar, a practice that is not clinically appropriate.^23^

From a patient perspective, one potential barrier to sustained biosimilar use is the nocebo effect. Nocebo effect, which is the process of patients experiencing new or worsening symptoms and adverse effects caused by their negative expectations and not the pharmacologic action of the medication itself, has been noted in several studies.^24,25^ Regardless of a lack of objective data to confirm disease worsening, the nocebo effect can lead to high rates of biosimilar discontinuation. Strategies to combat the nocebo effect include presenting information with confidence and focusing on positive attributes, providing a delivery of balanced information on the benefit-risk profile, and promoting shared decision making to encourage patient empowerment.^25^

Finally, legal actions around patents have led to significant delays in the biosimilar launch. Most notably in the utilization of adalimumab biosimilars, patent protection has limited access until 2023.^26^ The FDA and the Federal Trade Commission have partnered to help fight against anti-competitive business practices [FDA/FTC].

Ongoing advocacy efforts and partnerships between institutions, patients, payors, manufacturers, and regulators are critical to promote biosimilar adoption and uptake. Only then will the full potential of biosimilars be fully experienced by patients and the United States health care system.

## CONCLUSIONS

Current data support the efficacy and safety of biosimilars for the treatment of IBD; however, barriers to biosimilar adoption and use continue to be present. By creating and implementing a streamlined process that involves key stakeholders, these barriers can be addressed to implement biosimilar adoption and utilization, as successfully showcased by 2 institutions within the United States. On an organizational and legislative level, multiple steps and advocacy efforts are being undertaken to help promote the growth of biosimilar use.

## Data Availability

No new data were created or analyzed in the production of this manuscript.

## Notes

Financial Disclosure: None.
